# Supplementary material for: Gastric motility and pulmonary function in children with functional abdominal pain disorders and asthma: A pathophysiological study
Source: PLoS One. 2022 Jan 4;17(1):e0262086. doi: 10.1371/journal.pone.0262086 (PMC8726504; doi:10.1371/journal.pone.0262086)
Supplement: Attachment — Submitted filename: Answers to comments of reviewers R1.docx [file pone.0262086.s002.docx]

**Answers to comments of reviewers**

**Reviewer - 1**

Before approving this paper for publication, I suggest that the authors modify the discussion and clarify the following sections to avoid confusion.

Comment:
1) Why does more severe impairment of gastric motility in children suffering from both asthma and FAPDs, compared to either disease alone further strengthen the association of gut dysmotility and asthma? (page 18, lines 289-291, and repeated in Conclusion, page 21)

Answer:

Although this statement says there is impairment of gastric motility in children suffering from both asthma and FAPDs compared to either disease alone, it is not actually statistically significant. According to statistical analysis and results (Table 2), gastric motility is impaired significantly in children suffering from both diseases compared to control group (p<0.0001) and compared to children with asthma alone group (p<0.05) but not compared to children with FAPDs alone group (p>0.05). Therefore the above statement was removed from results section, discussion and conclusion of manuscript.

Comment:
2) How do the authors explain the fact that no correlation was documented between motility index and FEV1/FVC ratio or FEF 25-75% in asthmatic children (Results, page 16, paragraph 1), although the subjects did have impaired gastric motility (especially in the context of a positive correlation found among children with both FAPDs and asthma)? Please add comment to the Discussion section.

Answer:

Though it is observed a positive correlation between gastric motility parameters and lung function parameters (FEV1/FVC ratio or FEF 25-75%) in children having both diseases, we could not detect such correlation in children with only asthma and children with only FAPDs. This is because there could be either impairment of airway smooth muscle function at subclinical state in children in children with only FAPDS or or impairment in gastric motility is not severe enough in children with only asthma. Ttherefore, the degree of impairment is not strong enough to give such correlation for them to be associated each other. However, with the diseases progression, at one point, once the smooth muscle dysfunction in lung and gut become severe enough to have a strong correlation, then there is a possibility for these two disorders to get associated with each other sharing the same pathophysiological mechanism.

Comment: :
3) Is the suggested shared pathophysiological mechanism regarding smooth muscle impairment restricted to children with both diseases, or does subclinical pathology (either respiratory or gastrointestinal) exist in all cases? If yes, what would be the reason for inconsistent correlation results among different study groups?

Answer:

Though a positive correlation is observed between gastric motility and lung function parameters (FEV1/FVC ratio or FEF 25-75%) in children having both diseases, we could not detect such correlation in children with only asthma and children with only FAPDs. This may be because there could be either impairment of airway smooth muscle function or gastric motility function at subclinical state in children with only asthma or in children with only FAPDs, therefore, the degree of impairment is not strong enough to give such correlation for them to be associated each other. However, with the diseases progression, at one point, once the smooth muscle dysfunction in lung and gut become severe enough to have a strong correlation, then there is a possibility for these two disorders to get associated with each other sharing the same pathophysiological mechanism. Therefore, we suggest the possibility that smooth muscle impairment may exist at subclinical state either lung or gut in children with asthma or FAPDs

Comment:
4) The assertion of the conclusion needs toning down/adjustment accordingly to these revisions. Alternatively, the authors should include more information that clarifies and justifies their composition of the conclusion.

Answer

Revision was made accordingly in the conclusion according to the reviewer comment.

**Reviewer – 2**

Comment 1

Dear author, your research and idea of research are really interesting. Unfortunately, this paper is written in bad English and needs proofreading.

Answer

English was edited and proofreading was done again

Comment 2

Also, I partially disagree with the statement in the conclusion - gastrointestinal dysmotility is a possible common pathophysiological mechanism between these two diseases - as gastrointestinal dysmotility cannot really be a part of asthma pathomechanism... Later, it is well said: therefore, these diseases could arise as a result of a primary disruption of smooth muscle activity in the airways and gastrointestinal wall, which could be a possible pathophysiological mechanism for this association between asthma and FAPDs. I suggest you remove or rephrase the first part.

Also, I noticed that you use the same abbreviation for gastric emptying rate and gastroesophageal reflux, which can be confusing.

Answer

According to the reviewer, this statement (gastrointestinal dysmotility is a possible common pathophysiological mechanism between these two diseases) was removed from discussion and conclusion. The abbreviation is also changed.
