# Supplementary material for: Gastric motility and pulmonary function in children with functional abdominal pain disorders and asthma: A pathophysiological study
Source: PLoS One. 2022 Jan 4;17(1):e0262086. doi: 10.1371/journal.pone.0262086 (PMC8726504; doi:10.1371/journal.pone.0262086)
Supplement: Attachment — Submitted filename: Reviewer comments and asnwers R2.docx [file pone.0262086.s003.docx]

**Gastric motility and pulmonary function in children with functional abdominal pain disorders and asthma: a pathophysiological study**

Reviewer #1:

Comment:

In the revised manuscript, the authors repeat the statement: “gastric dysmotility is more severe in children having asthma+FAPDs than in having one of those diseases” (Discussion section - line 285), although they acknowledged this is not true (authors’ answer to my Comment 1)

Answer:

This is revised according to the reviewer comment

Comment:
Unfortunately, the authors failed to offer a plausible explanation/answer to my comments 2 and 3. Their conclusions, incorporated in the Discussion section, do not appear sound for several reasons. Specifically, they assume that muscle dysfunction is milder in asthma alone and FAPDs alone than in asthma+FAPDs, but the presented data (Table 2) does not support this assumption. They also suggest that disease progression and the degree of muscle impairment are important for a correlation to exist. They failed to relate to any reference that would back up their point of view. Not being able to find a plausible explanation for a study result is better than offering unsupported or confusing opinions.

Answer:

This is revised according to the reviewer comment

Concerning the Reviewer 2 comment – the authors again use the same abbreviation GER for two conditions, what is disappointing. (for example, see lines 290-295).

Therefore, I do not support publishing the work in this form. I encourage the authors to revise the Manuscript once again, mainly the Discussion, following this comment and the previous review comments.

Answer:

This is revised according to the reviewer comment
